# Laparoscopic Heller Myotomy for Symptomatic Epiphrenic Diverticula: A Modern Technique

**DOI:** 10.7759/cureus.83227

**Published:** 2025-04-29

**Authors:** Zoi Nitsa, Stylianos Faltsetas, Spyridon Davakis, Dimitrios Patsouras, Marianthi Vatrika, Alexandros Charalabopoulos

**Affiliations:** 1 1st Department of Surgery, Laiko General Hospital, National and Kapodistrian University of Athens, Athens, GRC; 2 1st Department of Surgery, University of Athens, Athens, GRC; 3 2nd Department of Surgery, Laiko General Hospital, National and Kapodistrian University of Athens, Athens, GRC

**Keywords:** achalasia, epiphrenic diverticulum, laparoscopic approach, laparoscopic myotomy, poem

## Abstract

Epiphrenic diverticula are one of the three types of esophageal diverticula, along with Zenker's and midesophageal, and are extremely rare. They are almost always associated with underlying esophageal motility disorders such as achalasia, diffuse esophageal spasm, and nutcracker esophagus. They are false diverticula and are found slightly above the cardio-esophageal junction on the right side. Symptoms usually appear in the digestive system and include dysphagia, regurgitation, and chest pain, as well as in the respiratory system with the occurrence of chronic cough and aspiration pneumonia. Not all epiphrenic diverticula are symptomatic, and treatment is indicated only when symptoms develop. Imaging methods, such as barium swallow, aid in diagnosing epiphrenic diverticula but should always be combined with endoscopy to exclude malignancy, since, rarely, epiphrenic diverticula are associated with squamous cell carcinoma. Manometry is crucial for confirming the presence of a motility disorder, as well as pH monitoring for symptomatic reflux. The treatment of the pathophysiologic basis of the genesis of the epiphrenic diverticula is the primary concern, and this involves surgical myotomy, as well as endoscopic techniques such as peroral endoscopic myotomy (POEM). The results of symptom remission after the surgical approach are high.

## Introduction

In the 1960s, Belsey's [1] and Effler et al.'s [2] pioneering works established that epiphrenic diverticula often coexist with esophageal motility disorders, challenging the notion that diverticulectomy could serve as a standalone treatment and bringing myotomy into focus as an alternative therapeutic option. Esophageal motor disorders occur when there is a lack of coordinated movement between the muscles of the lower esophageal sphincter (LES) and the lower esophagus, resulting in increased pressures within the esophagus. This elevated pressure can lead to the prolapse of the mucosa and submucosa through a weakness in the muscular layer, forming a pseudodiverticulum [3].

The typical sites where hernias or diverticula can develop are at the entry points of the nerves and blood vessels of the lower esophagus, which are naturally weaker areas [4]. Conditions commonly associated with epiphrenic diverticula include esophageal achalasia, diffuse esophageal spasm, and nutcracker esophagus, affecting more than three-quarters of patients with epiphrenic diverticula [5-8].

Surgical or endoscopic intervention is typically reserved for symptomatic patients, as asymptomatic cases can often be managed conservatively. However, special attention must be given to asymptomatic patients, especially during procedures such as blind intubation, due to an increased risk of diverticular injury and perforation [9].

## Case presentation

An 81-year-old man presented to the upper GI surgery department with a five-year history of chronic dysphagia, vomiting, and recurrent episodes of aspiration. These symptoms prompted a series of laboratory, imaging, and endoscopic evaluations, which revealed the presence of three epiphrenic diverticula and type 2 achalasia. Esophageal dilatation was noted to be mild. The diverticula were located 5 cm above the esophagogastric junction (EGJ). Given the clinical findings, the patient was diagnosed with epiphrenic diverticula in association with achalasia.

After considering the available options, the decision was made to proceed with laparoscopic Heller myotomy using a five-port technique as the treatment for achalasia. This approach was chosen given the absence of significant reflux disease in the patient, making fundoplication unnecessary.

Surgical approach

The patient was placed in a supine position with the upper and lower limbs in abduction in a reverse Trendelenburg position. The lead surgeon stood between the patient's legs, while the assistant positioned themselves on the left side of the patient. The monitor was positioned above the patient's head to ensure optimal visualization during the procedure.

Pneumoperitoneum was established via the open Hasson technique at the sub-umbilical site, with a pressure of 12 mmHg. The visual trocar was inserted sub-umbilically, while the two manipulation trocars were placed on the right and left sub-costal areas, above the umbilicus. The fourth assistant trocar was positioned 5 cm laterally to the surgeon's left manipulation trocar at the left sub-costal region. Additionally, a Nathanson retractor was inserted through the subxiphoid trocar for optimal liver retraction.

The dissection was performed with minimal invasion to preserve the physiological and anatomical anti-reflux mechanisms of the esophagus. The approach focused primarily on the anterior surface of the phrenogastric membrane, ensuring that the anterior vagus nerve and its branches were preserved throughout the procedure. The dissection extended to a point just beneath the pulmonary veins.

The myotomy incision was initiated on the esophagus as this provided a lower risk of mucosal injury, compared to performing a gastric myotomy. It is essential during myotomy to ensure that the instrument slides between muscle fibers and the mucosa, elevating the fibers away from the mucosal layer before performing the cut.

The esophageal myotomy extended for 7 cm, while the gastric myotomy was limited to 2 cm. Given that the dissection was kept to a minimum and no significant gastroesophageal reflux was identified, fundoplication was deemed unnecessary. This approach aligns with findings from multiple studies, which show no statistically significant difference in long-term outcomes regarding gastroesophageal reflux when fundoplication is omitted in such cases.

Postoperative outcome

The patient tolerated the procedure well, with no immediate complications. The postoperative course was uneventful, and the patient's symptoms, including dysphagia and regurgitation, significantly improved during follow-up visits. At six months post surgery, the patient reported a complete resolution of symptoms, with no further episodes of aspiration or difficulty swallowing (Video 1).

**Video 1:** Laparoscopic Heller myotomy Minimal Heller myotomy without fundoplication for three epiphrenic diverticula in an achalasia patient by laparoscopic approach

On the same day of the surgery, the patient began clear fluid intake without experiencing any symptoms of dysphagia. He tolerated the fluids well, with no signs of regurgitation or discomfort. The patient was discharged on the second postoperative day in stable condition, following a smooth recovery.

During the two-month follow-up examination, the patient reported significant improvement in his symptoms. He was able to eat without difficulty and had no further episodes of vomiting or aspiration. At the six-month follow-up, the patient continued to show remarkable improvement, maintaining a good nutritional status and no longer experiencing any symptoms of dysphagia, vomiting, or aspiration. He was well-fed and able to resume normal daily activities without limitations.

These follow-up results demonstrate the success of the procedure in addressing the patient's symptoms and improving his quality of life.

## Discussion

Epiphrenic diverticula have a prevalence of about 0.02%-3%. They are false diverticula, involving the last 10 cm of the esophagus, and most often occur 4-8 cm above the cardio-esophageal junction. Even though the risk of squamous cell carcinoma is low, about 0.6%, endoscopy should always be a part of the diagnostic algorithm [6]. Through the years, diverticulectomy was the standard treatment, but a deeper understanding of the pathophysiology showed that myotomy-based procedures should be the primary surgical approach [1,2]. Our case aligns with the literature demonstrating that addressing the underlying motility disorder through laparoscopic Heller myotomy can lead to substantial symptomatic relief without necessitating diverticulectomy. We should also add that we did not perform a diverticulectomy because the patient was 81 years old, and although he did not have any remarkable comorbidities, the thoracoscopic excision would have added extra risk, potentially outweighing the benefits of such a procedure.

Diverticulectomies carry high mortality and morbidity rates and should be considered only as a last resort in symptomatic patients, where previous conservative and surgical treatments have failed. Laparoscopic or endoscopic myotomy is considered the gold standard in the treatment of epiphrenic esophageal diverticula due to the motor dysfunction of the organ. The technique of surgical myotomy remains the same as for the treatment of achalasia, while the technique of endoscopy remains debatable in terms of length, depth, and the orientation it should have [9].

In 2019, Westcott et al. reported a case study of 22 patients with epiphrenic diverticula who had been treated by laparoscopic Heller myotomy and partial fundoplication, reducing significantly the need for diverticulectomy as the second stage in these patients [10]. Three years later, a literature review study reported similar results, supporting myotomy with fundoplication as the first-line treatment of epiphrenic diverticula [11,12].

## Conclusions

In cases where epiphrenic diverticulum coexists with the mobility disorder of the esophagus, diverticulectomy is not always necessary. In these patients, the first stage of treatment for the underlying condition is surgical myotomy or peroral endoscopic myotomy (POEM) in cases of esophageal achalasia, and in many cases, this is sufficient for the resolution of symptoms. Diverticulectomies are complex surgical procedures with an increased risk of complications, while other parameters such as the patient's age, comorbidities, and the improvement or not of symptoms after the treatment of the underlying pathology must be taken into account before applying them to patients.
